# The Impact Of Oral Health On Psychological Distress Among Older Adults In California

**DOI:** 10.1093/geroni/igab046.3144

**Published:** 2021-12-17

**Authors:** Esmeralda Melgoza, Jennifer Archuleta, Emma Tran, Gabino Abarca, Anna Fiastro

**Affiliations:** 1 UCLA Fielding School of Public Health, Los Angeles, California, United States; 2 UCLA, UCLA, Los Angeles, California, United States; 3 University of California, Los Angeles, Los Angeles, California, United States

## Abstract

In California, the population of adults ages 65 and older is projected to increase from 11% in 2010 to 19% in 2030. The aging of the population requires modifications in public health to ensure that people are not only living longer, but also healthier lives. Oral health is an important, but often overlooked factor that affects the overall health of older adults. Poor oral health increases the risk of physical comorbidities, decreases chewing performance, limits food choices, and exacerbates weight loss. Furthermore, poor oral health disrupts social health via decreases in verbal communication and facial expressions, such as smiling. This study examines the effects of oral health, assessed by tooth condition, on psychological distress among adults ages 65 and older in California. The study uses the 2019 California Health Interview Survey (CHIS), an annual survey of a representative sample of the state’s residential, noninstitutionalized population. Logistic regression models are used to determine the association between tooth condition on psychological distress controlling for gender, race, and elderly poverty index. Worsening tooth condition increases the odds of having psychological distress with lower odds among individuals 85+, and higher odds among women, and non-Hispanic whites compared to Hispanics. Public health programs and interventions are required in California to prevent and mitigate the impacts of poor oral health on psychological distress among the increasing and diverse older adult population.

